# A Lack of Parasitic Reduction in the Obligate Parasitic Green Alga *Helicosporidium*

**DOI:** 10.1371/journal.pgen.1004355

**Published:** 2014-05-08

**Authors:** Jean-François Pombert, Nicolas Achille Blouin, Chris Lane, Drion Boucias, Patrick J. Keeling

**Affiliations:** 1 Canadian Institute for Advanced Research, Department of Botany, University of British Columbia, Vancouver, British Columbia, Canada; 2 Department of Biological Sciences, University of Rhode Island, Kingston, Rhode Island, United States of America; 3 Entomology and Nematology Department, University of Florida, Gainesville, Florida, United States of America; Duke University Medical Center, United States of America

## Abstract

The evolution of an obligate parasitic lifestyle is often associated with genomic reduction, in particular with the loss of functions associated with increasing host-dependence. This is evident in many parasites, but perhaps the most extreme transitions are from free-living autotrophic algae to obligate parasites. The best-known examples of this are the apicomplexans such as *Plasmodium*, which evolved from algae with red secondary plastids. However, an analogous transition also took place independently in the Helicosporidia, where an obligate parasite of animals with an intracellular infection mechanism evolved from algae with green primary plastids. We characterised the nuclear genome of *Helicosporidium* to compare its transition to parasitism with that of apicomplexans. The *Helicosporidium* genome is small and compact, even by comparison with the relatively small genomes of the closely related green algae *Chlorella* and *Coccomyxa*, but at the functional level we find almost no evidence for reduction. Nearly all ancestral metabolic functions are retained, with the single major exception of photosynthesis, and even here reduction is not complete. The great majority of genes for light-harvesting complexes, photosystems, and pigment biosynthesis have been lost, but those for other photosynthesis-related functions, such as Calvin cycle, are retained. Rather than loss of whole function categories, the predominant reductive force in the *Helicosporidium* genome is a contraction of gene family complexity, but even here most losses affect families associated with genome maintenance and expression, not functions associated with host-dependence. Other gene families appear to have expanded in response to parasitism, in particular chitinases, including those predicted to digest the chitinous barriers of the insect host or remodel the cell wall of *Helicosporidium*. Overall, the *Helicosporidium* genome presents a fascinating picture of the early stages of a transition from free-living autotroph to parasitic heterotroph where host-independence has been unexpectedly preserved.

## Author Summary

*Helicosporidium* is a highly-adapted obligate parasite of animals. Its evolutionary origins were unclear for almost a century, but molecular analysis ultimately and surprisingly showed that it is a green alga, which means it has undergone an evolutionary transition from autotrophy to parasitism comparable to that of the malaria parasite *Plasmodium* and its relatives. Such transitions are often associated with the loss of biological functions that are no longer necessary in their novel environment and with the development of molecular mechanisms, sometimes quite sophisticated, to invade and take advantage of their hosts. Yet, very little is actually known about the early stages of the transition of a free-living organism to an obligate intracellular parasite. Here we sequenced the genome and transcriptome of *Helicosporidium*, and use it to show that the outcome of this transition is quite different from that of *Plasmodium*.

## Introduction

Helicosporidia are parasitic protists characterized by mature discoid cysts each containing a single filamentous and three ovoid cells [1], [2]. These parasites invade their invertebrate hosts *per os* and initiate their replicative stage within the digestive tract [3], [4]. The cysts, triggered by chemical changes in the gut, dehisce and release both the ovoid cells and filament cell. The ovoid cells remain in the gut lumen whereas the uncoiled and barbed filamentous cells penetrate the peritrophic membrane and become anchored to the host midgut cells. Over time the filamentous cells migrate through the midgut epithelium, breach the basement membrane, and invade the hemocoel. In the hemocoel the filament cells will transition to a vegetative stage that replicates by autosporulation; a select number at the four-cell stage will differentiate to the infectious cyst stage characterized by the three ovoid and a single filament cell [5], [6]. Unlike many parasites the vegetative cells of Helicosporidia can be cultured readily on defined media with limited nutrients, suggesting that despite being a parasitic species, they have retained a diverse slate of metabolic pathways allowing for saprobic growth.

The evolutionary origin of Helicosporidia remained uncertain for nearly 100 years since their initial description, although various characters were used to suggest some relationship with microsporidian, sporozoan, and myxosporidian parasites. Recently, however, ultrastructural observations surprisingly revealed that the vegetative state of *Helicosporidium* cells is similar to that of the achlorophylous trebouxiophyte green alga *Prototheca* [1], and subsequent phylogenetic inferences derived from actin/tubulin and plastid sequences strongly confirmed this affiliation [7], [8]. The discovery that Helicosporidia are trebouxiophycean green algae raises some interesting questions about the evolution of parasitism: within this single lineage are found free-living autotrophs like most other green algae, but also a variety of symbiotic species, opportunistic pathogens, and perhaps even obligate intracellular parasites, all of which diversified within a relatively narrow evolutionary timescale. The transformation from free-living to parasitic lifestyles often includes the shedding of metabolic functions that are no longer required as the parasite relies increasingly on its host for energy and nutrients [9]. The parasitic relationship may be opportunistic at first, but can switch to being obligate upon reaching a certain threshold of host-dependence, after which the formerly free-living organism can no longer revert to its previous lifestyle due to the ratchet-like nature of these losses. We do not often think of photosynthetic organisms with progenitors for parasitic ones, but a variety or parasitic lineages had at one time photosynthetic ancestors, including oomycetes, several dinoflagellates, and most famously the apicomplexan parasites such as the malaria parasite, *Plasmodium* (see [10], [11] and references therein). One of the first things to be lost in photosynthetic species is presumably their ability to harvest energy from light and fix carbon. Harnessing light from within large-bodied hosts is probably very difficult if not impossible, and the resulting metabolic deficit must lead to a significant shift in the balance between the host and parasite. Some of these lineages (e.g. oomycetes) probably evolved through a heterotrophic intermediate, but others possibly began their association with animals as phototrophs. How the transformation to parasites took place is of great interest, but unfortunately because it happened so long ago (around 1 bya for *Plasmodium* [12]) and is now so complete, the critical early stages have long been wiped away. Helicosporidia, in contrast, appear to have evolved from free-living autotrophs relatively recently [13], [14], and might therefore provide some interesting insights. Fossils records and molecular clock analyses suggest that Trebouxiophytes as a group arose in the early Neoproterozoic [13], from which the trebouxiophycean subgroup Chorellales later emerged around 100 million years ago (mya) [14]. Both *Helicosporidium* and the non-photosynthetic trebouxiophycean *Prototheca* arose from within the Chorellales [13], so the adaption to parasitism in Helicosporidia occurred less than 100 mya.

To specifically investigate how the metabolic and proteomic complexity of pathogenic Helicosporidia are distinguished from their free-living and symbiotic trebouxiophycean relatives, we sequenced the genome and transcriptome of *Helicosporidium* sp. ATCC50920, a parasite of the black fly *Simulium jonesi* [1], [15]. We show that the *Helicosporidium* genome is 2.5-fold smaller than genomes from the free-living and symbiotic trebouxiophytes, *Coccomyxa subellipsoidea* C-169 [16] and *Chlorella variabilis* NC64A [17], which are themselves extremely small for trebouxiophyte genomes. However, the reduction of the *Helicosporidium* genome is not tied to a massive reduction in metabolic functions: despite its small genome size and parasitic nature, it surprisingly still encodes all major metabolic pathways, with the exception of a small number specifically related to photosynthesis. Even here, the reduction is not complete: all genes relating to light harvesting and electron transport are missing, but the *Helicosporidium* carbon fixation pathway is nearly complete but for the lack of ribulose-1, 5-bisphosphate carboxylase/oxygenase (RuBisCO) and a pyruvate kinase. The smaller size of the *Helicosporidium* genome can be attributed to a greater degree of genome compaction (e.g. fewer and smaller introns, and smaller intergenic regions), and most significantly to a lower complexity of gene families, particularly those related to DNA packaging/replication pathways. We also show that the gene family complexity of other metabolic pathways has increased, in particular relating to chitin metabolism, which likely represented a key development in the ability of *Helicosporidium* to develop in the insect host. Overall, these results give our first view into the early stage in the transition from a free-living autotroph to an obligate pathogen.

## Results

### General features of the *Helicosporidium* draft genome

**Figure 1:**
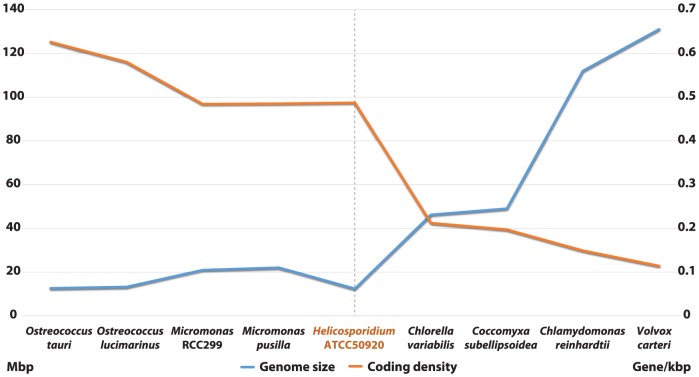
Inversed correlation between size and coding density in sequenced green algal genomes. Assembled sizes (Mbp) and coding densities (gene/kbp) are shown on the left and right Y-axis, respectively.

**Figure 2:**
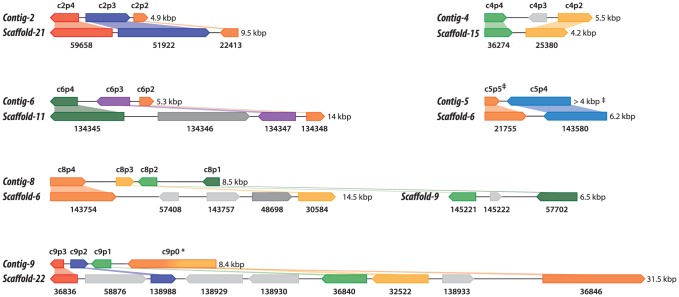
Conserved gene clusters between the *Helicosporidium *and *Chlorella* genomes. Only syntenic clusters from the ten largest *Helicosporidium* contigs are shown. Genes from *Helicosporidium* are shown on top; *Chlorella* genes are shown below. Locus_tag prefixes (*Helicosporidium*, H632_; *Chlorella*, CHLNDRAFT_) were omitted for clarity (see Data S2 for PFAM product names). The corresponding contigs (*Helicosporidium*) or scaffolds (*Chlorella*) are indicated on the left; in the *Chlorella* scaffolds, adjacent genes are not always labelled incrementally. Genes that are absent from the other genome are colored in light gray. Genes that have been relocated are shown in dark gray. Partial genes are indicated by double daggers (‡). In *Helicosporidium* the c9p0 gene, indicated by an asterisk, is predicted as a single entity encompassing the *Chlorella* 32522 and 36846 genes.

Shotgun Illumina reads of total DNA were assembled into 11,717 contigs totalling 13,684,556 bp (62.2% GC). Contamination filters suggest a maximum of 1% overall contamination, located in the smaller-sized contigs. Removal of the mitochondrial and plastid genomes and filtering of the small contigs resulted in 5,666 contigs of at least 500 bp in size (12,373,820 bp total; N50 3,036 bp, 61.7% GC), with an average coverage of 62× (Table 1). Based on the current data, we estimate the *Helicosporidium* genome at a maximum size of 17±0.5 Mbp. This corresponds well to a genome size estimate of 13 Mbp, derived from karyotype visualisation by clamped homogeneous electrical field (CHEF) electrophoresis [18]. A total of 6,035 protein-encoding genes were predicted among the assembled 12.4 Mbp, with an average of 2.3 exons (366 bp/exon) and 1.3 introns (168 bp/intron) per gene. Coding density in the *Helicosporidium* genome is high (0.487 gene/kb) compared to the free-living and symbiotic trebouxiophytes *Coccomyxa* (0.197 gene/kb) and *Chlorella* (0.212 gene/kb), but lower than that of the 12 Mbp genomes of the picoplanktonic prasinophycean green algae in the genus *Ostreococcus* (0.626 and 0.580 gene/kb; Table 1, Figure 1). Identifiable transposable elements are rare in the assembled *Helicosporidium* contigs, although micro- and minisatellites and regions of generally low complexity were found (Data S1). Comparing the genomic assemblies with the transcriptome revealed a total of 95.4% of the genes attributed to known metabolic pathways (Table S1) were found in both datasets, and only 3.6% were found exclusively in the transcriptome. This correlated well with the overall percentages of transcriptomic contigs mapping on the genomic ones (≥1000 bp; 92.3%, ≥1500 bp; 95.9%), suggesting that the total coding potential of *Helicosporidium* is well-represented in the draft genome. The *Helicosporidium* genome shares little gene order conservation with the other green algal genomes: only 30% of the genes located within its ten largest contigs are arrayed in syntenic clusters with those of *Chlorella* (Figure 2), with no apparent metabolic relationship between the genes present in these clusters.

**Table 1:** Main features of sequenced green algal genomes.

	*Ostreococcus tauri*	*Ostreococcus lucimarinus*	*Micromonas RCC299*	*Micromonas pusilla*	*Helicosporidium ATCC50920*	*Chlorella variabilis*	*Coccomyxa subellipsoidea*	*Chlamydomonas reinhardtii*	*Volvox carteri*
Chromosomes	20	21	17	19	10	12	20	17	19
Size (Mbp)^a^	12.6	13.2	20.9	22	12.4 (17)	46.2	49	112	131
GC (%)	59	60	64	65	62	67	53	64	56
Genes	7,892	7,651	10,109	10,672	6,035	9,791	9,629	16,709	14,971
Gene density^b^	0.626	0.580	0.484	0.485	0.487	0.212	0.197	0.149	0.114
Av. exon/gene	1.6	1.3	1.6	1.9	2.3	7.3	8.2	7.4	7.8
Av. exon size	750	970	958	730	366	170	157	240	236
Av. intron size	126	187	163	193	168	209	273	336	500
Rel. version	2.0	2.0	3.0	3.0	1.0	1.0	2.0	4.0	2.0

a Assembled sizes. The *Helicosporidium* estimated size is indicated between parentheses.

b Gene density values (gene per kbp) were calculated from the assembled size.

### A surprisingly near-complete metabolic profile

The *Helicosporidium* genome is small: it is approximately 2.5 times smaller than the two other complete trebouxiophyte genomes, *Coccomyxa* and *Chlorella* (Table 1, Figure 1), which are themselves at the extremely low end of the spectrum of estimated genome sizes in this lineage (Figure S1). But this small size is not a reflection of a severe reduction in metabolic potential. Indeed, the *Helicosporidium* genome encodes almost all of the major biological functions that are shared between the genomes of its trebouxiophycean relatives and that of the chlorophycean green alga *Chlamydomonas reinhardtii* (Table S1).

**Figure 3:**
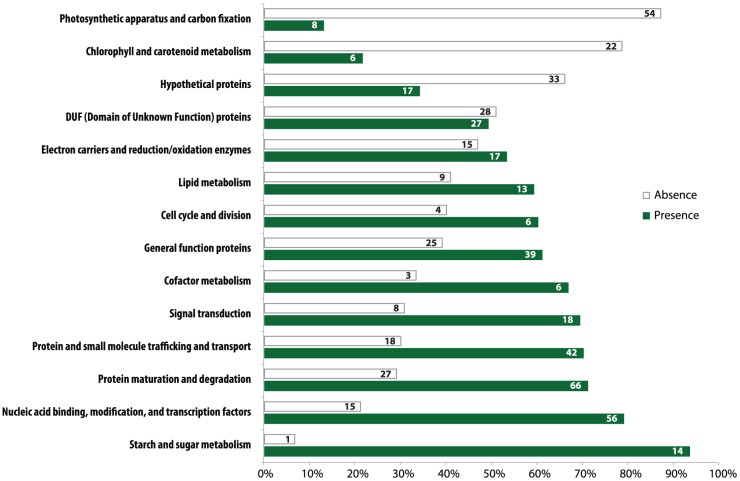
Distribution of GreenCut2 proteins in *Helicosporidium*. The pathways indicated on the left are from GreenCut2 [19]. Numbers inside the white and green bars indicate the total number of genes that are absent or present, respectively, from the corresponding pathway in the *Helicosporidium* genome. The bars are drawn to scale according to the respective percentage of genes that have been retained (green) or lost (white) in the corresponding pathway.

**Figure 4:**
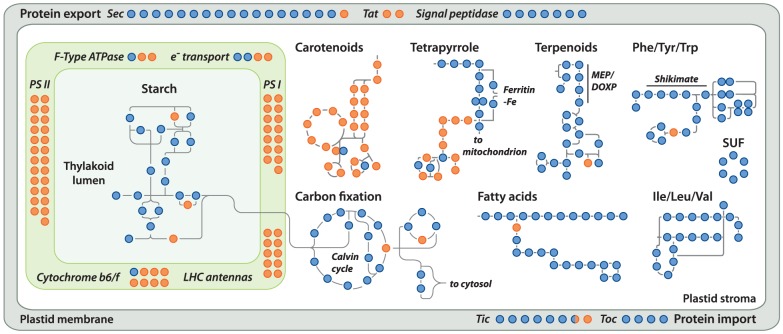
Localization of *Helicosporidium* losses amongst the major plastid metabolic pathways. Proteins present and absent in *Helicosporidium* are shown in blue and orange, respectively. The metabolic pathways shown here have been simplified for visualization; only the proteins present in other green algae were considered and signal peptidases from the protein export pathway perform their functions in the cytosol. The KEGG orthology (ko) identifiers [50], [51] are: carbon fixation, ko00710; carotenoids, ko00906; fatty acids, ko01040; ile/leu/val, ko00290; phe/tyr/trp, ko00400; protein export, ko03060; starch, ko00500; terpenoids, ko00900; tetrapyrrole, ko00860 (Table S1). SUF (sulfur mobilization) is not present in KEGG. Tat; Twin-Arginine Translocation pathway, Tic/Toc; Translocon at the Inner/Outer envelope membrane of Chloroplasts.

Gene loss in the *Helicosporidium* genome is significantly concentrated in photosynthesis-related pathways, and even here gene loss is surprisingly sparse given its non-photosynthetic, parasitic nature. The *Helicosporidium* genome encodes 56% of the plastid-targeted proteins predicted by the GreenCut2 database [19] (Figure 3), whereas both the photosynthetic *Coccomyxa* and *Chlorella* encode 96% of these proteins. The overall distribution of these losses in plastid metabolism is not random, but is concentrated on processes related to light-harvesting (Figure 4, Data S3, S4, S5, S6, S7, S8, S9, S10, S11). The heme synthesis branch of the tetrapyrrole pathway is complete in *Helicosporidium*, but the branch leading to the biogenesis of chlorophyll has been lost (Figures 4 and S2, Data S3, S4, S5, S6, S7, S8, S9, S10, S11). Similarly, *Helicosporidium* cannot synthesize carotenoids. It does not encode light-harvesting antenna proteins and photosystems I and II are completely absent, which parallels the loss of all photosynthesis-related genes in its plastid genome [20]. Surprisingly however, the *Helicosporidium* genome has retained an almost complete carbon fixation pathway despite lacking two major components *rbcL/rbcS* coding respectively for the large and small subunits of the ribulose-1,5-bisphosphate carboxylase oxygenase (RuBisCO) and *ppdK*, a pyruvate orthophosphate dikinase involved in pyruvate interconversions in the C_4_ pathway (Figure 4). Similarly, *Helicosporidium* has retained some proteins involved in electron transport and components of the F-type ATPase and cytochrome b6f (Figure 4). Starch and fatty acid metabolic pathways are more or less intact, as is the terpenoid biosynthesis pathway and its isoprenoid non-mevalonate MEP/DOXP synthesis branch. The SUF iron-sulfur cluster biosynthetic pathway is conserved as well, alongside its ISC/NIF mitochondrial counterpart [21], [22]. Not surprisingly the protein import and export systems are intact.

Outside the plastid, *Helicosporidium* metabolism shows little signs of significant reduction. The *Helicosporidium* genome encodes all proteins required for the biosynthesis of conventional aminoacyl-tRNAs, except selenocysteine. Despite its conservation across the green algae, we found no evidence for the presence of a selenocysteine synthase in the genomic and transcriptomic *Helicosporidium* datasets. The o-phosphoseryl-tRNA(sec) kinase also required for selenocystenyl-tRNA synthesis is missing too, however all other enzymes involved in the metabolism of selenocompounds are present. *Helicosporidium* appears incapable of endogenous RNA interference and, like the picoeukaryotes *Ostreococcus tauri* and *Ostreococcus lucimarinus*, lacks the genes coding for the Dicer and Argonaute proteins. These genes are found in single copies in the *Chlorella* and *Coccomyxa* genomes whereas three paralogous copies of DC1 and AGO1 are found in the *Chlamydomonas* genome [23]. In *Chlamydomonas*, this expanded set has been postulated to mediate the silencing of its numerous transposable elements [24]. The few losses observed in the remaining *Helicosporidium* pathways are either palliated by bypass enzymes or affect the synthesis or homeostasis of uncommon metabolites (Table S2).

**Figure 5:**
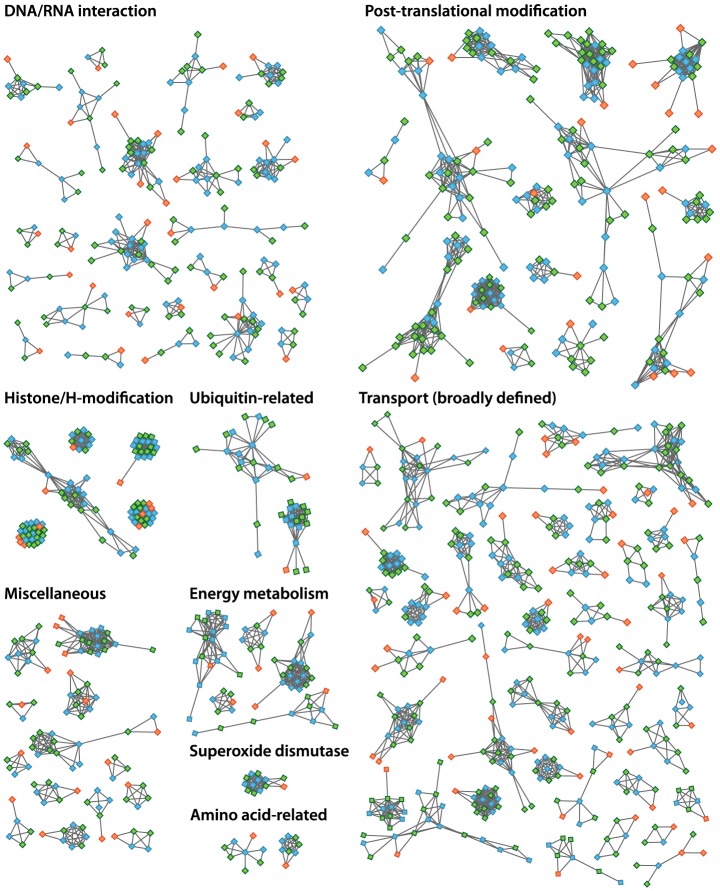
Evolutionary gene network analysis showing functional contractions in *Helicosporidium* relative to *Chlorella* and *Coccomyxa*. The connected components depicted here represent a small portion of the genes involved in each of these functional categories. *Helicosporidium*, *Coccomyxa* and *Chlorella* are represented by orange, blue and green nodes, respectively. Interactions between the different components are denoted by gray lines connecting the nodes.

The increased level of compaction and loss of photosynthetic genes in the *Helicosporidium* genome cannot explain its 2.5-fold reduction in genome size: other significant differences in gene content exist between the parasite and its free-living relatives. Given that *Helicosporidium*, *Coccomyxa*, and *Chlorella* were found to encode almost the same overall functional categories of genes in common with other green algae (Table S1), one possibility is that *Helicosporidium* possess fewer and/or smaller gene families. To investigate the complexity of gene families, we compared the *Helicosporidium*, *Chlorella*, and *Coccomyxa* predicted proteomes via an evolutionary gene network analysis [25]. A total of 100 connected components, excluding photosynthesis-related products, were found to exhibit a lower representation in *Helicosporidium* compared with its free-living relatives. These were manually curated into 9 functional categories based on annotation of the three gene sets (Tables S3 and S4, Figure 5). Interestingly, the functional categories where *Helicosporidium* has a reduced gene family complexity are for the most part not what are broadly defined as ‘operational’ genes where reduction that might be related to increased dependence on the host. Instead, the most drastic reductions in *Helicosporidium* are gene families in functional groups that are correlated with the size and complexity of the genome. The few exceptions are in amino acid and some other metabolic families, but most of the reduction relates to genes involved in chromosome packing, transcription, translation, post-translational modification, and protein turnover. Most surprisingly, *Helicosporidium* has not seen an increase in the complexity of transporter families, which might be expected of a parasite as host dependence grows; instead, this functional class is most reduced in *Helicosporidium* compared with *Coccomyxa* and *Chlorella*.

### Gene family expansions: Chitinases

Taking an opposing view, to what gene families may have expanded due to the adaptation to parasitism, revealed a single obvious expansion of functional significance. A total of 14 genes with putative glycosyl hydrolase (GH) activity were identified throughout the *Helicosporidium* genome and were also found within its transcriptome (Table 2). All of these proteins appear to belong to the GH18 chitinase family, whereas plant chitinases normally come from the GH19 family. The *Chlorella* genome encodes two GH18 and one GH19 chitinase, and these are assumed to be involved in the remodelling of its cell wall, which has been experimentally demonstrated to contain chitin [17], [26]. The GH19 chitinase in *Chlorella* was acquired by horizontal gene transfer from a large DNA virus [17] and is not found in either the *Helicosporidium* or *Coccomyxa* genomes. Conversely, the *Chlorella* GH18 chitinases are found across the green algae. In *Helicosporidium*, the extra 12 copies appear to have been generated by recent duplication events. We found no evidence for the transfer of genetic material from insects in the *Helicosporidium* genome or transcriptome. A total of 13 of the 14 *Helicosporidium* chitinases contain the Dx_2_DxDxE/P motif essential for chitinolytic activity. Insect and bacterial chitinases with experimentally confirmed catalytic activity also contain one or more of three additional motifs: Kx_6_GG, MxYDx(x)G, and Gx_3_Wx_2_DxD [27]–[29]. Thirteen of the chitinases in *Helicosporidium* contain one or two of these additional motifs and demonstrate high conservation in their orientation (Table 2, Figure S3). However, in addition to H632_c1867p1 which appears to lack this domain, three show substitutions within the Dx_2_DxDxE motif and may be therefore be inactive.

**Table 2:** Summary statistics for predicted chitinase proteins in *Helicosporidium* genome.

	Catalytic, binding, turnover domains			Conserved insect chitinase motifs							
Protein	Gly18	Cht BD	PEST	Dx_2_DxDxE	Kx_6_GG	MxYDxG	Gx_3_Wx_2_DxD	SP signal	Met	Length	kDa
H632_c1356p0	+	−	+	+	+	+	−	−	+	323	35.57
H632_c269p0	+	−	−	+	+	−	−	−	−	288	31.72
H632_c3129p0	+	−	−	+	+	+	−	−	−	223	23.72
H632_c3224p0	+	−	−	+	+	+	−	−	−	222	23.79
H632_c1110p2	+	+	−	+	+	−	−	−	−	144	15.16
H632_c1418p0	+	+	+	+ D(3) to G	+	−	−	−	+	382	42.22
H632_c1867p1	+	+	−	−	+	−	−	−	+	324	34.5
H632_c2552p0	+	−	−	+ D(1) to T	+	−	−	−	+	266	29.42
H632_c3570p0	+	−	−	+ E to P	+	−	−	−	+	266	28.95
H632_c5208p0	+	−	−	+	+	−	−	−	+	185	20
H632_c855p0	+	+	+ +	+	+	−	−	+	+	437	47.06
H632_c982p0	+	−	−	+	+	−	−	−	+	195	20.77
H632_c3633p0	+	+ +	−	+	−	−	−	−	−	262	27.27
H632_c4362p0	+	−	−	+	+	−	−	−	−	182	19.85

Gly18: glycosyl hydrolase catalytic domain (SM00036), Cht BD: type II chitin-binding domain, PEST: proteolytic target site, SP signal: secretory pathway signal present, Met: methionine at position one in protein prediction, Length: peptide length, kDa: estimated weight of mature peptide.

Another class of proteins that might be expected to be relevant to the origin of parasitism in Helicosporidia are, unfortunately, the unidentified or ‘unique’ ORFs. To see whether these represented a high proportion of the genes in *Helicosporidium*, we identified predicted proteins of at least 100 amino acids that are not found in *Coccomyxa* and *Chlorella*, which resulted in 882 distinct proteins (Data S12). A small number of these proteins have clear or putative homologs in the *Volvox* and/or *Chlamydomonas* genomes but the vast majority are unique and have no known homologs. The few cases with homologues in *Volvox* and *Chlamydomonas* are sulfotransferases, glycosyltransferases or hydrolases with chitinase activity (as mentioned above), a 2-oxoglutarate Fe(II)-dependent oxygenase, and a cyclin. In contrast, the predicted proteins without known green algal homologs could not be assigned to any putative function in PFAM homology searches at an *E*-value cut-off of 1e-10. Five of these unknown proteins (H632_c233p3, H632_c338p0, H632_c531p0, H632_c1976p1, H632_c4072p0) display mid to low similarity with bacterial sequences of unknown function, but they are unambiguously encoded on contigs encoding clearly eukaryotic genes, and are therefore not bacterial contaminants. From the transcriptome, we can also conclude that the majority of these proteins are expressed, with 585 of the 882 found in transcriptome data at an *E*-value threshold of 1e-15.

### Horizontal gene transfer from/to viruses

**Figure 6:**
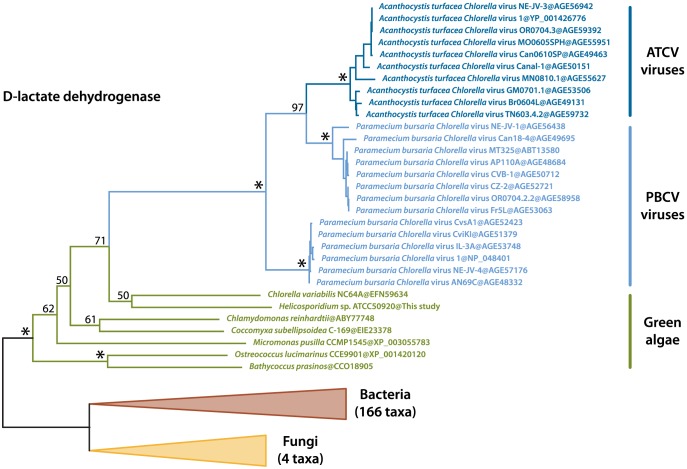
Maximum likelihood tree of the D-lactate dehydrogenase transferred by HGT between green algae and their viruses. The best protein ML tree is shown here. Bootstrap support is indicated above the corresponding nodes. Fungal and bacterial tips were converted to triangles for concision. ATCV, *Acanthocystis turfacea Chlorella* viruses; PBCV, *Paramecium bursaria Chlorella* viruses.

We identified two transcripts sharing a high identity with viral sequences (*E*-value threshold of 1e-40), the closest relatives being *Paramecium bursaria Chlorella* viruses (PBCV) and *Acanthocystis turfacea Chlorella* viruses (ATCV). These two transcripts were also identified in the *Helicosporidium* genomic contigs, and both are assembled with *Helicosporidium* nuclear genes and are therefore not the result of viral contamination. The first transcript (a374428r16) codes for a dUDP-D-glucose 4,6 dehydratase that is also found in the genomes of *Chlorella*, *Coccomyxa* and various other green algae, and has been reported as an example of host to virus horizontal gene transfer (HGT) [30], [31]. The second transcript (a28443r121) encodes a D-lactate-dehydrogenase and is also found across the green lineage. Phylogenetic analyses including the closest D-lactate-dehydrogenase sequences clusters the green algal sequences with homologues from nucleocytoplasmic large DNA viruses that infect them, with strong bootstrap support (Figure 6). Overall, this suggests D-lactate-dehydrogenase represents another case of host-virus HGT in the green algal lineage.

## Discussion

### A lack of loss in the evolution of parasitism

A recurring theme in the evolution of parasite genomes and parasitism in general is reduction. In parallel with the more constructive process of developing sometimes sophisticated mechanisms to invade and take advantage of their hosts, parasite evolution generally involves the selective pruning of biological functions that are no-longer mandatory to survival in the host. This reduction has occurred independently many times during the evolution of parasites, and is generally reflected in their genomes, streamlined sometimes solely by a loss of genes, but other times also by an overall shortening of coding and intergenic regions as well as a loss of introns, resulting in much smaller and compact genomes than those of their free-living relatives [9]. In very few cases have photosynthetic algae made this transition, but the famous exception is the apicomplexans, including the malaria parasite *Plasmodium*. Here the same process has taken place and although the plastid has been retained, it has been reduced to a cryptic form that lacks most of its ancestral metabolic pathways and has retained nothing whatsoever related to carbon metabolism.

*Helicosporidium* breaks from these trends in significant ways. With an almost perfect conservation of the core green algal metabolic pathways, its genome is small, but can hardly be considered reduced, which may reflect its relatively recent adaptation to parasitism. Particularly surprising is the retention of an almost complete pathway for carbon fixation. *Helicosporidium* has lost nearly all genes associated with light harvesting, photosystems, and chlorophyll biogenesis, so how it uses carbon fixation pathways is an interesting question. Carbohydrate storage, and in particular the use of starch could be the main driver behind the retention of carbon fixation genes. Successful parasites often sequester resources from their host [32] and converting simple sugar molecules to large starchy polymers polarizes the directionality of carbohydrate exchanges. Exactly how efficient are the *Helicosporidium* permeases at sugar uptake and other energy-related metabolites from the immediate environment is unclear; considering that this is one of the gene families of reduced complexity, but such sequestration may be a prerequisite for survival.

The coding density of the *Helicosporidium* genome is only marginally higher than its closest relatives, and almost on par with those of a number of free-living prasinophytes (Table 1; Figure 1). Green algal genome sizes range widely across lineages (Figure S1), and expansions as well as contractions are likely to have occurred several times independently. Accordingly, we cannot currently distinguish between a reduction of DNA packaging/replication/translation pathways in *Helicosporidium*, an expansion of these pathways in *Chlorella/Coccomyxa*, or a combination of both. A better phylogenetic framework as well a greater sampling depth of green algal nuclear genomes will be required to polarize the directionality of this event, but the reduced gene family complexity observed in picoprasinophytes [33]–[35] does argue in favour of an expansion from a lean ancestral state.

Despite its small size, the *Helicosporidium* genome does feature one small expansion. Chitinases are uniformly rare in green algae where genomic data are available, suggesting this gene family is ancestrally limited in the green algae (which fits with its likely function in remodelling a minor component of the cell wall in some species). The expansion of chitinases in *Helicosporidium* over its sister taxa *Chlorella* and *Coccomyxa* therefore likely represents a unique adaptation that directly resulted from or even contributed to its parasitic lifestyle in insect hosts. The *Helicosporidium* infection mechanism consists of the oral uptake of cysts, dehiscence in the midgut lumen, ingression through the midgut, and entry into the hemocoel where the organism multiplies. In a number of insect parasites, including the malarial parasite *Plasmodium*, the presence of exogenous chitinases from the GH18 family within the arthropod midgut tract is either mandatory for infection or associated with increased pathogenicity by allowing pathogens to pass through the peritrophic membrane [36], [37]. Disruption of chitinase activity with blocking agents interferes with the sporogonic development of malarial parasites, which is restored upon addition of exogenous chitinases [38]. One may speculate that the *Helicosporidium* chitinases serve multifunctional roles and operate at various stages *in vivo*. Potentially, chitinases sequestered in the cysts are activated by insect digestive proteases, and then digest the cyst wall to initiate the release of the invasive filament cell in the gut lumen. Alternatively, the chitinases released by the ovoid cells, may loosen the chitin matrix of the peritrophic membrane, allowing the ingress of the filamentous cell into the ectoperitrophic space. It is important to note that members of the GH18, in addition to binding to and digesting the chitin can also target various GlcNAc-containing glycans that comprise various exocellular matrices including insect basement membranes. Binding to such substrates may aid in the establishment of infection on the hemocoel associated tissues. Finally the production of these enzymes could soften the exoskeleton, which could play a role in the egress of the infectious cyst stage from diseased insects.

## Materials and Methods

### Tissue culture and DNA/RNA purification

Vegetative cells of the *Helicosporidium* sp. (ATCC50920) a parasite of the black fly *Simulium jonesi* [1], [15] was propagated in stationary cultures of sabouraud dextrose broth for five days at 27°C. Cells were harvested by centrifugation (5,000 rcf for 10 min) and pellets resuspended in a minimal volume sterile H_2_O and used for nucleic acid extraction. For genomic DNA preparation, a total of 2×10^9^ cells were suspended in the yeast lysis buffer (Epicentre Biotechnologies, Madison WI) and homogenized with Bead Beater technology. The extracted nucleic acid phase was treated with DNase free RNase and subjected to chloroform phenol extraction, precipitated with ethanol, and suspended in TE buffer. A total of 14.1 µg high molecular weight DNA were recovered and submitted for sequencing. For total RNA extraction the resuspended harvested cells were immediately added to liquid N_2_ and ground with mortar and pestle to break the outer pellicles. The resulting frozen cell powders were processed initially with TRizol Reagent then processed with Purelink RNA Mini kit (Ambion). Column eluants were treated with RNase-free DNase and analysed with the 2100 Bioanalyzer (Agilent Technologies, Inc). Samples (10 µg) producing RIN values of 8.9 to 9.2 were selected for subsequent sequence analysis (see below).

### DNA and RNA sequencing

Total DNA and RNA from *Helicosporidium* sp. ATCC 50920 (mitochondrial, plastid and nuclear) were sequenced by Fasteris SA (Plan-les-Ouates, Switzerland) using the Illumina platform. Two independent DNA sequencing runs were performed. In the first, 18,618,066 reads (54-bp paired ends; 323-bp inserts; average standard deviation, 19) totaling 1,005,375,564 bases were sequenced with the Illumina GA-IIx platform and the Chrysalis 36 cycles v 4.0 sequencing kit. In the second, 17,110,904 reads (51-bp paired ends; 241-bp inserts; average standard deviation, 64) totaling 872,656,104 bases were sequenced with the Illumina HiSeq 2000 platform and the TruSeq chemistry. Total RNA was sequenced using the Illumina directional mRNA-SEQ protocol with the Illumina HiSeq 2000 platform and the TruSeq chemistry. A total of 83,075,963 reads (100-bp single ends) were generated (8,307,596,300 bases total). Read quality for each Illumina data set was assessed with FastQC (version 0.10.1; Babraham Bioinformatics, Babraham Institute [http://www.bioinformatics.babraham.ac.uk]).

### Genome assembly

Paired-end reads were assembled *de novo* with Ray [39] 2.0.0 rc8 using iterative k-mer values of 21 to 31 on 8 processing cores (2 Intel Xeon E5506 CPUs at 2.13 GHz) with a maximum RAM allowance of 96 Gb. The resulting contigs were filtered by size with sort_contigs.pl (Advanced Center for Genome Technology, University of Oklahoma [www.genome.ou.edu/informatics.html]), and contigs shorter than 500 bp were discarded. The contigs of at least 500 bp were conserved for downstream analyses. The 500+-bp contigs were used as canvas to generate a BLAST [40] database with MAKEBLASTDB from the NCBI BLAST 2.2.26 package, the mitochondrial and plastid contigs were identified by BLAST homology searches using the mitochondrial (GenBank accession number NC_017841, [41]) and plastid (GenBank accession number NC_008100, [20]) genomes as queries, and separated from the nuclear contigs. Putative contaminants were assessed by homology searches against the NCBI non-redundant database.

### Transcriptome assembly

RNA-Seq reads were filtered using a sliding-window quality approach with Sickle (Bioinformatics Core, University of California, Davis [https://github.com/najoshi/sickle]) under the default parameters, and the overall read quality reassessed after filtering with FastQC. Illumina adapter sequences were then removed from the filtered sequences using custom Perl scripts, and PolyA-tails were removed from the reads with TrimEST from the EMBOSS [42] 6.4.0 package. The filtered transcriptome reads were assembled with Trinity's Inchworm module with a maximum RAM allowance of 90 Gb (–JM 90G) on 8 processing cores (2 Intel Xeon E5506 CPUs at 2.13 GHz). Contigs were filtered by size with sort_contigs.pl and contigs of at least 250 bp were selected for downstream analyses. Transcriptomic contigs were mapped on the genomic ones with GMAP version 2014-01-21 [43] using the default parameters.

### Genome annotation

The nuclear contigs of at least 500-bp in length were sorted by size and renumbered incrementally using customs Perl scripts. Contigs were then processed with the Maker 2.11 annotation gauntlet [44], [45] using the *Chlorella* gene model as implemented in Augustus 2.5.5 [46]. The resulting GFF annotations files were processed, curated, and converted to GenBank annotations files using custom Perl scripts. Putative functions were assigned using homology searches against the PFAM database (*E*-value threshold of 1E-30; Table S5). Transposable elements were searched for with RepeatMasker [http://repeatmasker.org] using Repbase version 20130422 [47].

### Genome size estimation

Illumina reads from the mitochondrial and plastid genome were first filtered out from the total dataset with bowtie 0.12.9 [48] using –un and –al the flags against indexes built from the organelle sequences. Filtered nuclear reads were then mapped with bowtie against the 5,666 contigs (≥500 bp) with the –S flag, and the coverage estimated from the SAM file with Tablet 1.12.12.05 [49] and the coveragestat.py python script. The genome size was then estimated using the following formula: [# of reads X read length]/coverage.

### Pathways mapping and network analyses

KEGG metabolic pathway maps for the green algae *Chlamydomonas reinhardtii*, *Volvox carteri*, *Ostreococcus tauri* and *Ostreococcus lucimarinus* were retrieved from the KEGG pathway databases [50], [51], the proteins sorted accordingly, and then used as queries for homology searches against the *Helicosporidium*, *Chlorella* and *Coccomyxa* proteomic, genomic and transcriptomic datasets (the *Chlorella* and *Coccomyxa* data was retrieved from the JGI website). BLASTP and TBLASTN searches were performed using *E*-value thresholds of 1E-10 and 1E-05, respectively. Genes not found in searches against any of the three datasets were considered absent from the corresponding organism. Network analyses were performed according to [25]. Specifically, all possible edges were drawn between pairs of genes if their reciprocal BLASTP comparisons to one another met all of the following conditions: *E*-value<1E-10, minimal hit identity >20, at least 20% of the shortest gene's length had identical residues in the match, and the hit length >20 amino acids. The network was then filtered to include underrepresented *Helicosporidium* genes compared to *Coccomyxa* and *Chlorella*. Functional annotations for the genes comprising each connected component (GenBank, KOG, KEGG, Interpro [52], and Pfam) were used to characterize each connected component by its inferred biological function. Plastid-targeted proteins from GreenCut2's Table S2 [19] were extracted from the corresponding *Chlamydomonas reinhardtii* (version 3.1) and *Arabidopsis thaliana* (http://www.arabidopsis.org/) protein catalogs and converted to custom BLAST databases with MAKEBLASTDB from the NCBI BLAST package. *Helicosporidium*, *Chlorella* and *Coccomyxa* were searched independently against both GreenCut2 databases with BLASTP (proteins) and TBLASTN (genome and transcriptome) using *E*-value thresholds of 1E-10 and 1E-05, respectively.

### Chitinase identification

Putative glycosyl hydrolases identified in the *Helicosporidium* genome were annotated for catalytic and chitin binding domains using SMART 7 [53] and endo-proteolytic sites often located within developmental insect chitinases were identified with ePESTfind [54]. The glycosyl hydrolase catalytic domains were annotated manually for the presence and orientation of key amino acid motifs. Secretory signal motifs were searched for with TargetP 1.1 [55] and PredAlgo [56].

### Phylogenetic analyses

Amino acid sequences retrieved from GenBank were aligned with the L-INS-I algorithm from MAFFT 7.029b [57]. Phylogenetic models were selected with ProtTest 3.2 [58]. Maximum Likelihood phylogenetic reconstructions were performed with PHYML 3.0 [59] under the LG+Γ4+I model of amino acid substitution [60].

### Data deposition

The *Helicosporidium* data was deposited at DDBJ/EMBL/GenBank under NCBI BioProject ID PRJNA188927 and accession AYPS00000000. The version described in this paper is version AYPS01000000. The predicted proteins and RNAs are also available in Data S13 and S14, respectively. All custom Perl scripts are available on GitHub (https://github.com/JFP-Laboratory).

## Supporting Information

Data S1 Transposable elements found in the *Helicosporidium* contigs. Repeats were searched for with RepeatMasker [http://repeatmasker.org] using Repbase version 20130422.
(TXT)

Data S2 PFAM annotations of the Figure 2 proteins. The searches were performed with an *E*-value cut-off of 1.0; Figure 2 proteins that are absent from this file are hypothetical proteins.
(TXT)

Data S3 Orthology map of the valine/leucine/isoleucine biosynthesis pathway (ko00290) retrieved from KEGG [50], [51]. Genes that are present in *Helicosporidium* are indicated by yellow boxes. Genes that are absent from *Helicosporidium* but present in *Chlamydomonas* are indicated by red boxes. Genes that are absent from both *Helicosporidium* and *Chlamydomonas* are indicated by empty boxes.
(PNG)

Data S4 Orthology map of the phenylalanine/tyrosine/tryptophan biosynthesis pathway (ko00400) retrieved from KEGG [50], [51]. Genes that are present in *Helicosporidium* are indicated by yellow boxes. Genes that are absent from *Helicosporidium* but present in *Chlamydomonas* are indicated by red boxes. Genes that are absent from both *Helicosporidium* and *Chlamydomonas* are indicated by empty boxes.
(PNG)

Data S5 Orthology map of the starch and sucrose metabolism pathway (ko00500) retrieved from KEGG [50], [51]. Genes that are present in *Helicosporidium* are indicated by yellow boxes. Genes that are absent from *Helicosporidium* but present in *Chlamydomonas* are indicated by red boxes. Genes that are absent from both *Helicosporidium* and *Chlamydomonas* are indicated by empty boxes.
(PNG)

Data S6 Orthology map of the carbon fixation pathway in photosynthetic organisms (ko00710) retrieved from KEGG [50], [51]. Genes that are present in *Helicosporidium* are indicated by yellow boxes. Genes that are absent from *Helicosporidium* but present in *Chlamydomonas* are indicated by red boxes. Genes that are absent from both *Helicosporidium* and *Chlamydomonas* are indicated by empty boxes.
(PNG)

Data S7 Orthology map of the porphyrin and chlorophyll metabolism pathway (ko00860) retrieved from KEGG [50], [51]. Genes that are present in *Helicosporidium* are indicated by yellow boxes. Genes that are absent from *Helicosporidium* but present in *Chlamydomonas* are indicated by red boxes. Genes that are absent from both *Helicosporidium* and *Chlamydomonas* are indicated by empty boxes.
(PNG)

Data S8 Orthology map of the terpenoid backbone biosynthesis pathway (ko00900) retrieved from KEGG [50], [51]. Genes that are present in *Helicosporidium* are indicated by yellow boxes. Genes that are absent from *Helicosporidium* but present in *Chlamydomonas* are indicated by red boxes. Genes that are absent from both *Helicosporidium* and *Chlamydomonas* are indicated by empty boxes.
(PNG)

Data S9 Orthology map of the carotenoid biosynthesis pathway (ko00906) retrieved from KEGG [50], [51]. Genes that are present in *Helicosporidium* are indicated by yellow boxes. Genes that are absent from *Helicosporidium* but present in *Chlamydomonas* are indicated by red boxes. Genes that are absent from both *Helicosporidium* and *Chlamydomonas* are indicated by empty boxes.
(PNG)

Data S10 Orthology map of the unsaturated fatty acids biosynthesis pathway (ko01040) retrieved from KEGG [50], [51]. Genes that are present in *Helicosporidium* are indicated by yellow boxes. Genes that are absent from *Helicosporidium* but present in *Chlamydomonas* are indicated by red boxes. Genes that are absent from both *Helicosporidium* and *Chlamydomonas* are indicated by empty boxes.
(PNG)

Data S11 Orthology map of the protein export pathways (ko03060) retrieved from KEGG [50], [51]. Genes that are present in *Helicosporidium* are indicated by yellow boxes. Genes that are absent from *Helicosporidium* but present in *Chlamydomonas* are indicated by red boxes. Genes that are absent from both *Helicosporidium* and *Chlamydomonas* are indicated by empty boxes.
(PNG)

Data S12 *Helicosporidium* proteins that are not found in other trebouxiophytes. Predicted proteins of at least 100 amino acids that are not found in the trebouxiophytes *Chlorella* and *Coccomyxa* [*E*-value threshold 1E-05] are included in this file. The vast majority of these proteins are unique and have no known homologs.
(TXT)

Data S13 Predicted proteins in *Helicosporidium*. The proteins were predicted from the genomic contigs with MAKER 2.11 using the *Chlorella* gene model as implemented in Augustus 2.5.5.
(TXT)

Data S14 *Helicosporidium* RNA-Seq contigs. The *Helicosporidium* RNA-Seq data was assembled with Trinity's Inchworm module from quality filtered reads.
(FASTA)

Figure S1 Green algal genome sizes in the phylum Chlorophyta. Sequenced green algae are labelled in black with the source indicated between parentheses. Estimated values based on nuclear DNA content from Kapraun [61], [62] are color-coded according to their respective group. Blue, U, Ulvophyceae; Orange, C, Chlorophyceae; Green, T, Trebouxiophyceae; Purple, P, Prasinophyceae. Lower and upper estimates, when present, are shown in dark and light colors, respectively. Note that the real and estimated sizes of the *Ostreococcus tauri* genome differ by an order of magnitude.
(PDF)

Figure S2 Heme and chlorophyll pathways in *Helicosporidium*. Genes present in *Helicosporidium* are indicated in orange. Genes absent are shown in red. This simplified schema is derived from KEGG pathway KO00860 [50], [51].
(PDF)

Figure S3 CLUSTALW alignment of the *Helicosporidium* chitinase Gly18 catalytic domains. Conserved motifs are shown in blue. BLOSUM, Gap open penalty: 35, Gap extend penalty 0.75.
(TIFF)

Table S1 Distribution of the *Chlamydomonas reinhardtii* KEGG metabolic pathways in the trebouxiophytes *Helicosporidium*, *Chlorella* and *Coccomyxa*. The *Chlamydomonas*, *Chlorella* and *Coccomyxa* proteins and genomic/transcriptomic contigs are labelled according to the official headers provided in the JGI data (http://genome.jgi.doe.gov/).
(XLSX)

Table S2 Enzymes that are bypassed or lost in *Helicosporidium* non-photosynthetic pathways. The Dicer [K11592; EC:3.1.26.-] and Argonaute [K11596] proteins absent from *Helicosporidium* are not included in the table; the only KEGG pathway currently including Dicer/Argonaute proteins relates to cancer.
(XLSX)

Table S3 Underrepresented *Helicosporidium* metabolic pathways in evolutionary gene network analyses. The connected components represent a small portion of the genes involved in each of these functional categories.
(XLSX)

Table S4 InterProScan5 analyses of the connected components from Figure 5. *Helicosporidium*; sheet 1, *Chlorella*; sheet 2, *Coccomyxa*; sheet 3. The complete InterProScan5 format definition can be found at [https://code.google.com/p/interproscan/wiki/OutputFormats].
(XLSX)

Table S5 GenBank-annotated proteins in *Helicosporidium* sp. Only proteins displaying matches at an *E*-value cutoff of 1E-30 against PFAM were annotated for release in the GenBank database.
(XLSX)
