# Supplementary figures and images for: A Lack of Parasitic Reduction in the Obligate Parasitic Green Alga Helicosporidium
Source: PLoS Genet. 2014 May 8;10(5):e1004355. doi: 10.1371/journal.pgen.1004355 (PMC4014436; doi:10.1371/journal.pgen.1004355)

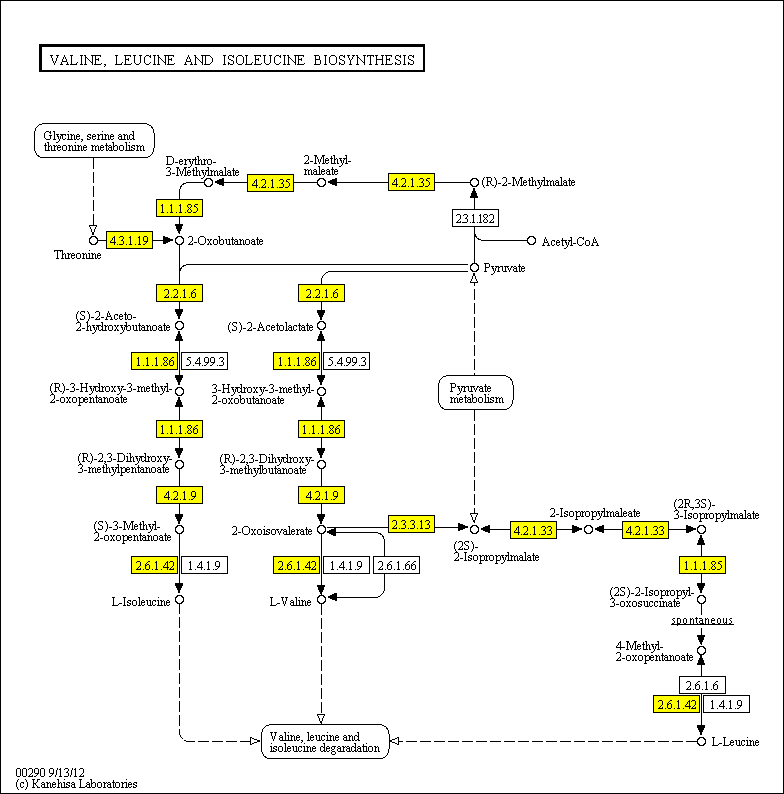

Supplement: Data S3 — Orthology map of the valine/leucine/isoleucine biosynthesis pathway (ko00290) retrieved from KEGG [50], [51]. Genes that are present in Helicosporidium are indicated by yellow boxes. Genes that are absent from Helicosporidium but present in Chlamydomonas are indicated by red boxes. Genes that are absent from both Helicosporidium and Chlamydomonas are indicated by empty boxes. (PNG) [file pgen.1004355.s003.png]

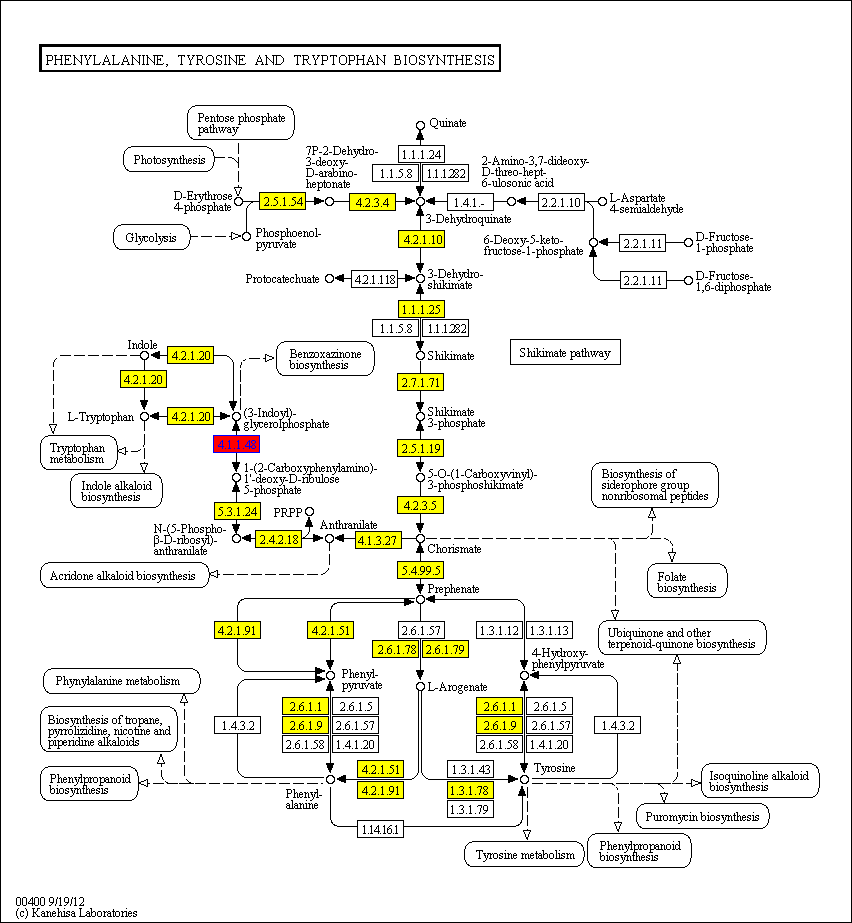

Supplement: Data S4 — Orthology map of the phenylalanine/tyrosine/tryptophan biosynthesis pathway (ko00400) retrieved from KEGG [50], [51]. Genes that are present in Helicosporidium are indicated by yellow boxes. Genes that are absent from Helicosporidium but present in Chlamydomonas are indicated by red boxes. Genes that are absent from both Helicosporidium and Chlamydomonas are indicated by empty boxes. (PNG) [file pgen.1004355.s004.png]

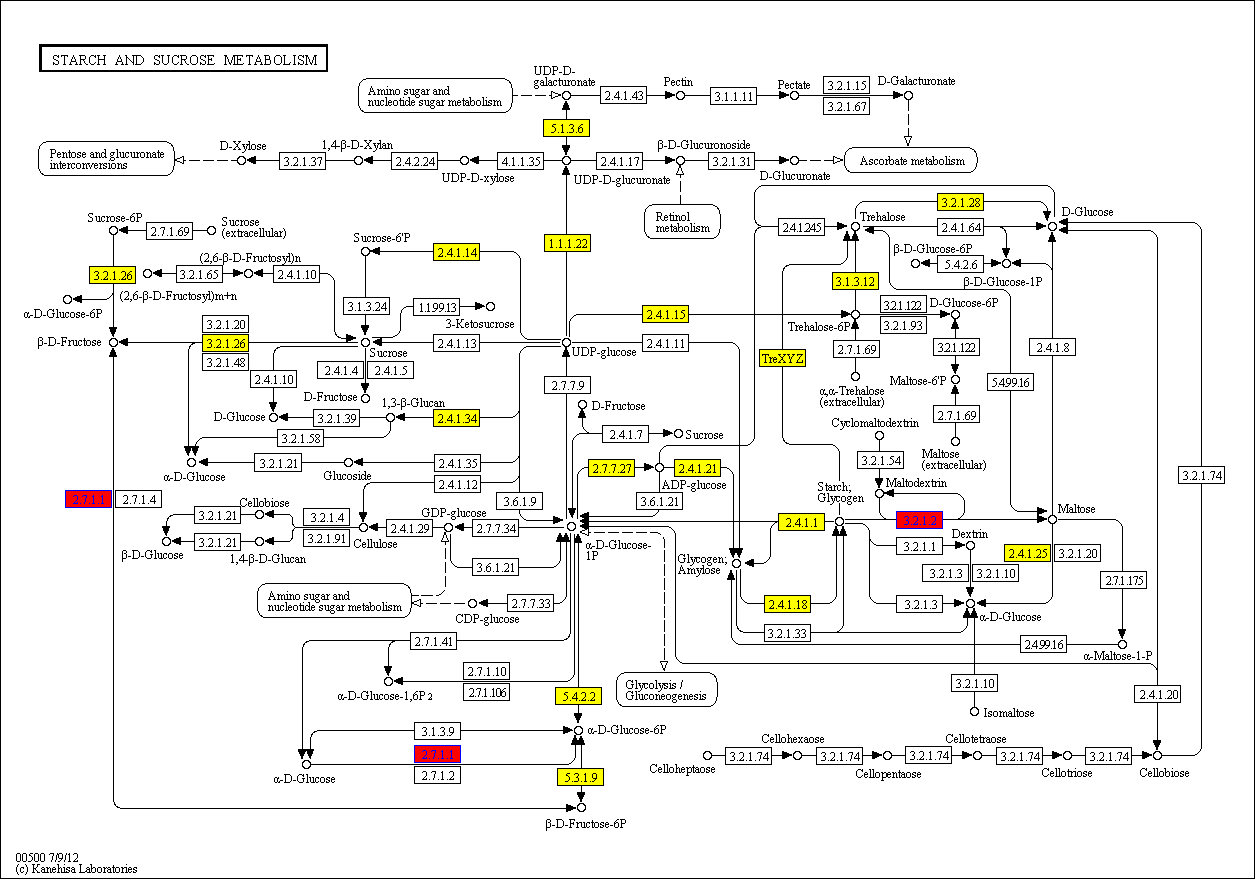

Supplement: Data S5 — Orthology map of the starch and sucrose metabolism pathway (ko00500) retrieved from KEGG [50], [51]. Genes that are present in Helicosporidium are indicated by yellow boxes. Genes that are absent from Helicosporidium but present in Chlamydomonas are indicated by red boxes. Genes that are absent from both Helicosporidium and Chlamydomonas are indicated by empty boxes. (PNG) [file pgen.1004355.s005.png]

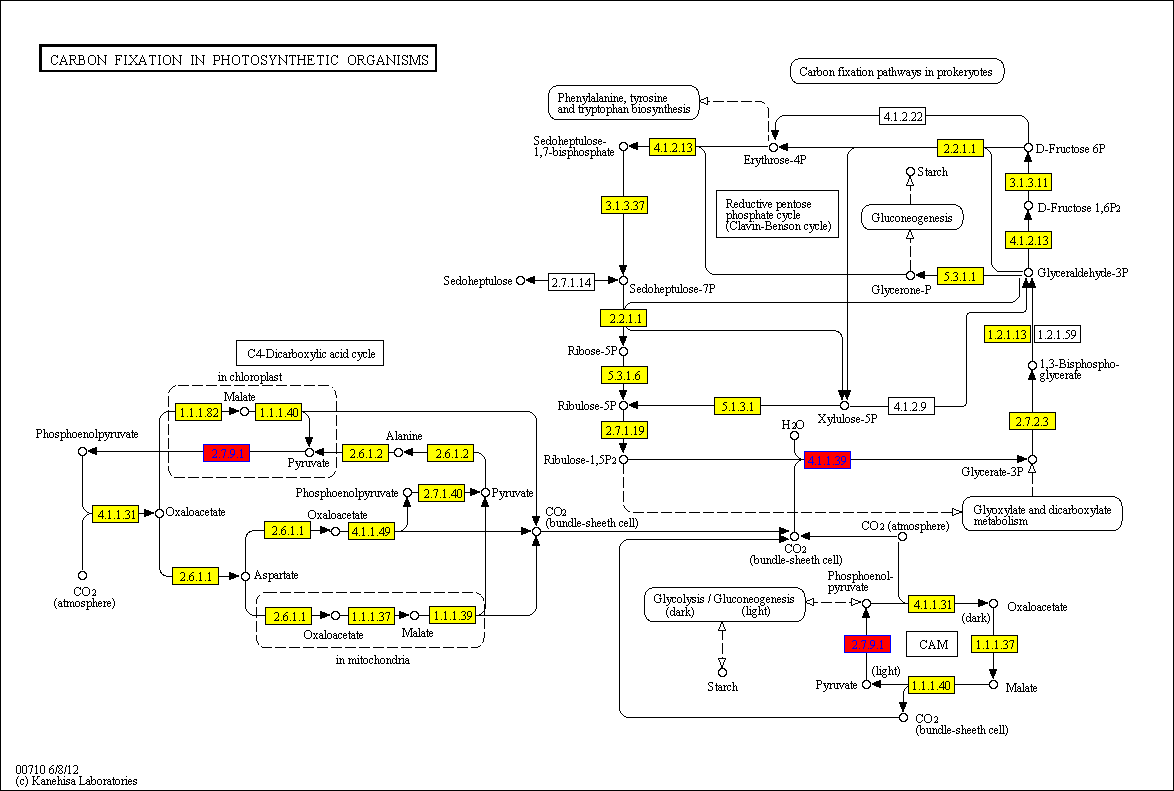

Supplement: Data S6 — Orthology map of the carbon fixation pathway in photosynthetic organisms (ko00710) retrieved from KEGG [50], [51]. Genes that are present in Helicosporidium are indicated by yellow boxes. Genes that are absent from Helicosporidium but present in Chlamydomonas are indicated by red boxes. Genes that are absent from both Helicosporidium and Chlamydomonas are indicated by empty boxes. (PNG) [file pgen.1004355.s006.png]

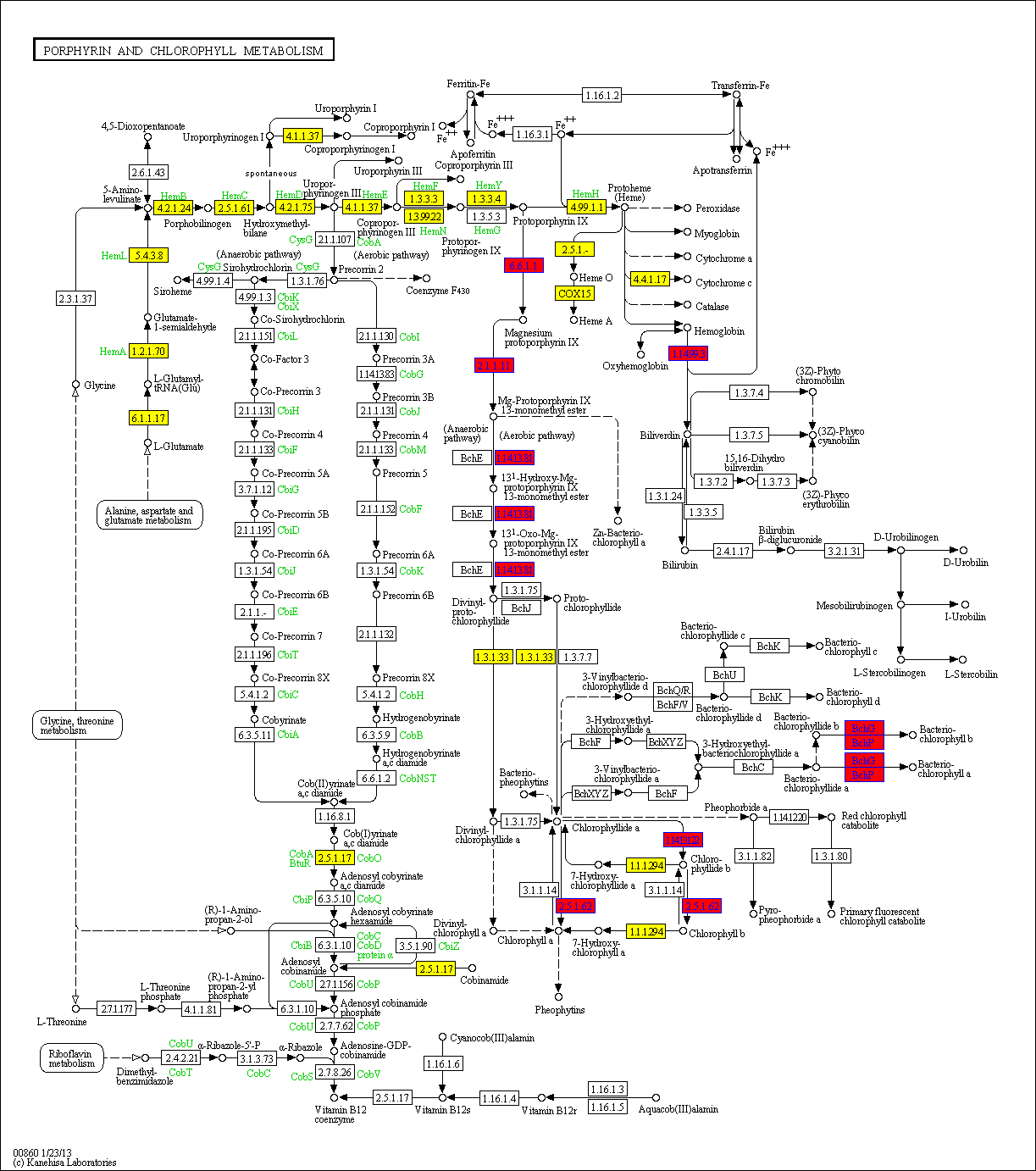

Supplement: Data S7 — Orthology map of the porphyrin and chlorophyll metabolism pathway (ko00860) retrieved from KEGG [50], [51]. Genes that are present in Helicosporidium are indicated by yellow boxes. Genes that are absent from Helicosporidium but present in Chlamydomonas are indicated by red boxes. Genes that are absent from both Helicosporidium and Chlamydomonas are indicated by empty boxes. (PNG) [file pgen.1004355.s007.png]

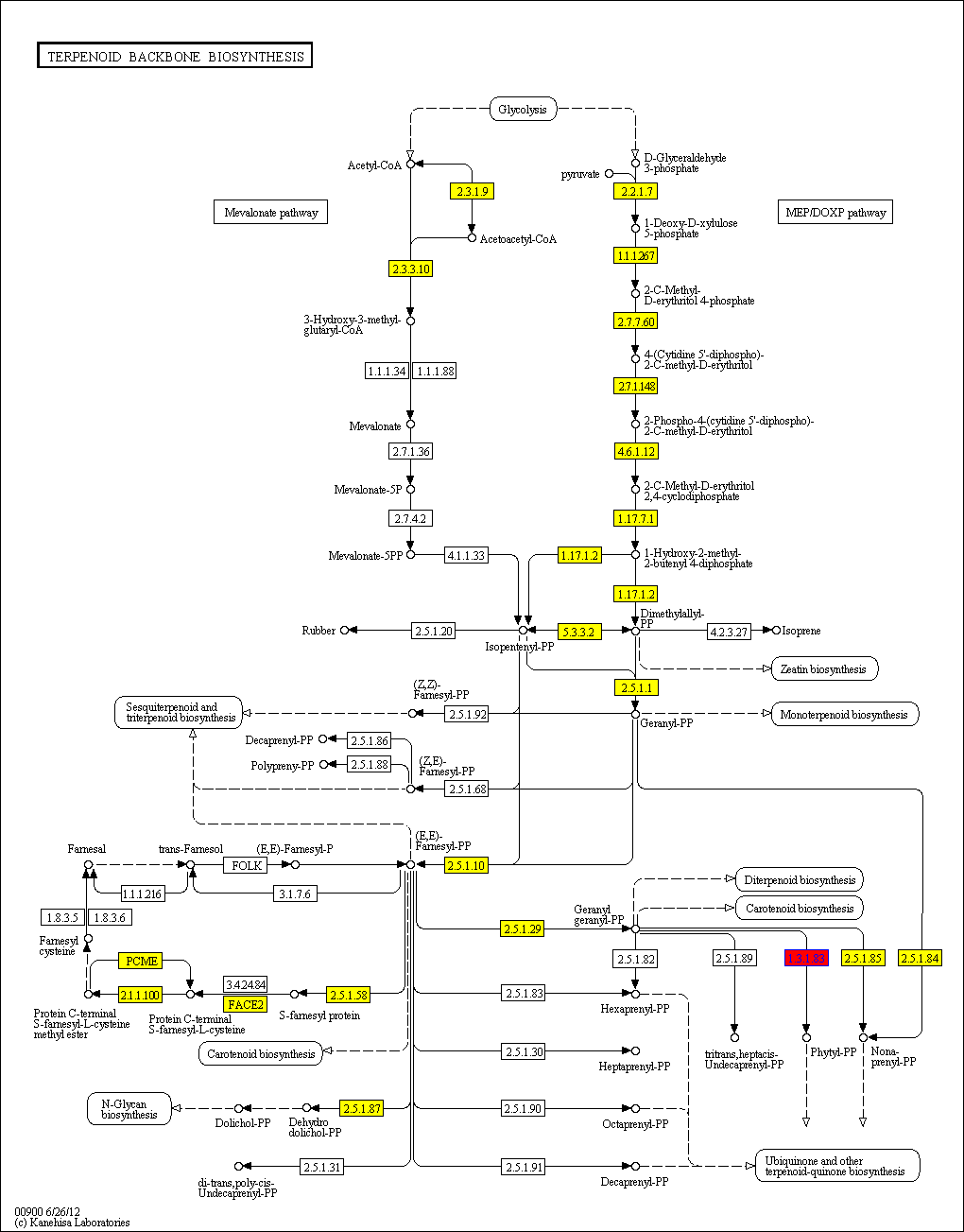

Supplement: Data S8 — Orthology map of the terpenoid backbone biosynthesis pathway (ko00900) retrieved from KEGG [50], [51]. Genes that are present in Helicosporidium are indicated by yellow boxes. Genes that are absent from Helicosporidium but present in Chlamydomonas are indicated by red boxes. Genes that are absent from both Helicosporidium and Chlamydomonas are indicated by empty boxes. (PNG) [file pgen.1004355.s008.png]

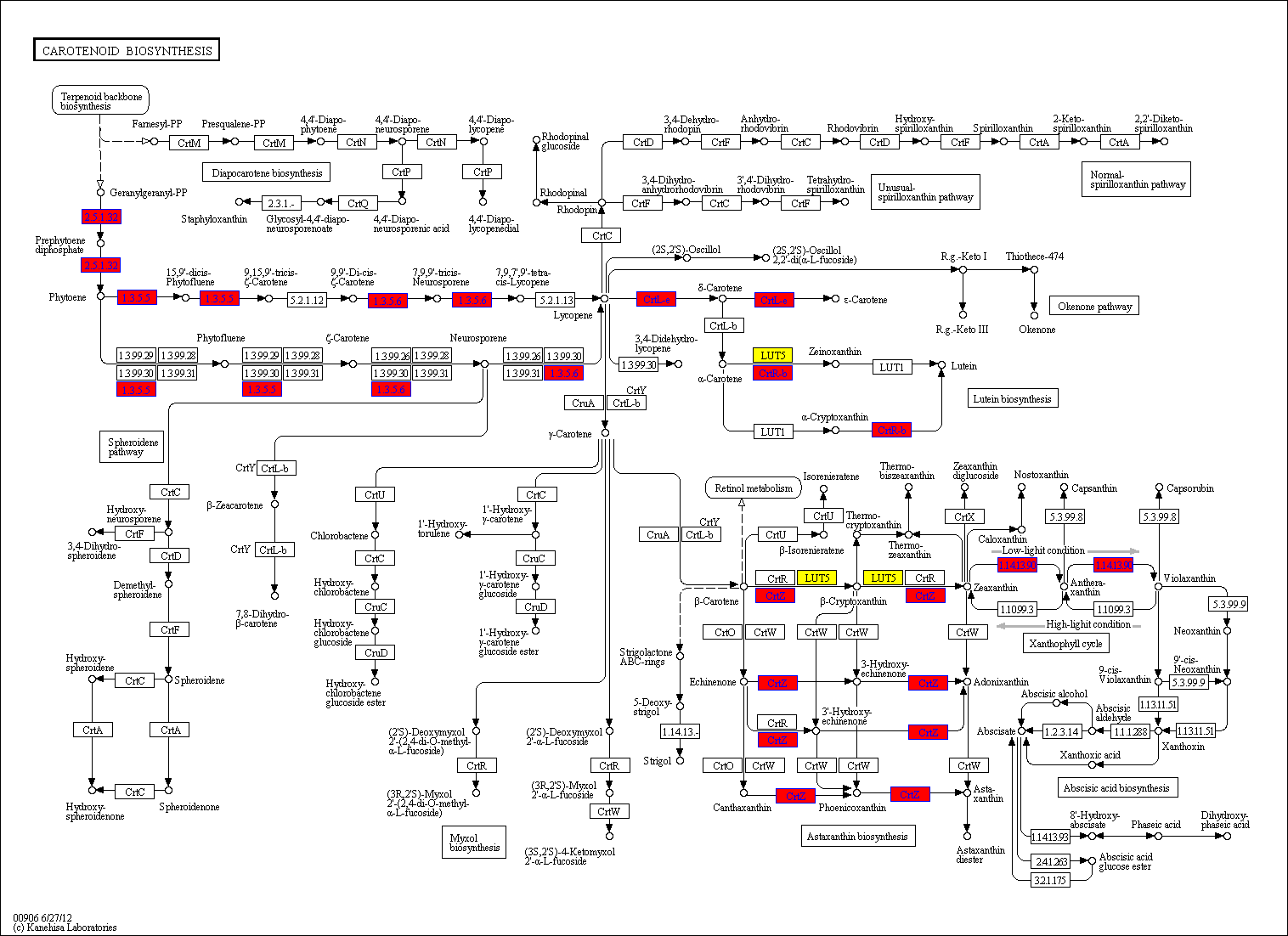

Supplement: Data S9 — Orthology map of the carotenoid biosynthesis pathway (ko00906) retrieved from KEGG [50], [51]. Genes that are present in Helicosporidium are indicated by yellow boxes. Genes that are absent from Helicosporidium but present in Chlamydomonas are indicated by red boxes. Genes that are absent from both Helicosporidium and Chlamydomonas are indicated by empty boxes. (PNG) [file pgen.1004355.s009.png]

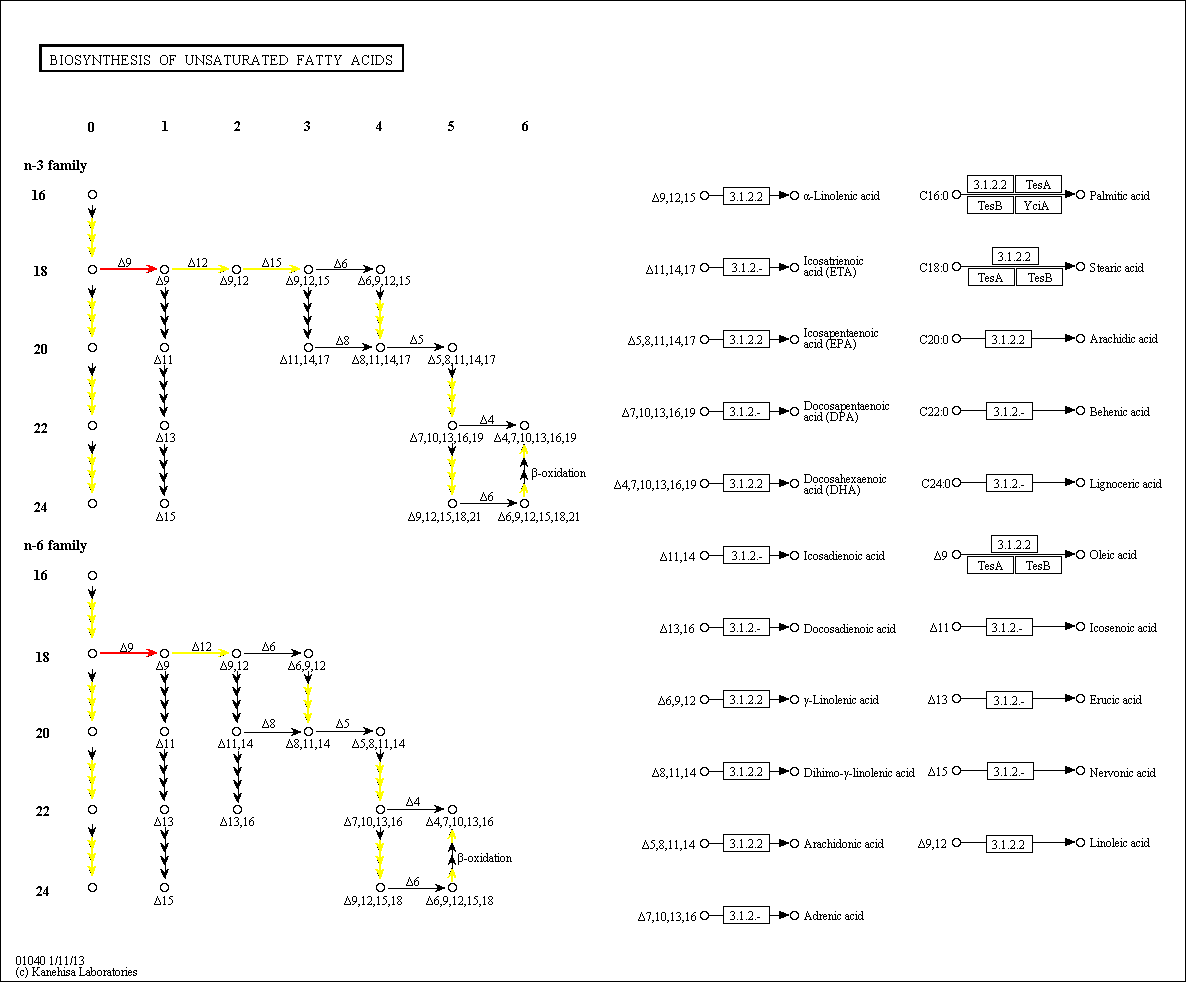

Supplement: Data S10 — Orthology map of the unsaturated fatty acids biosynthesis pathway (ko01040) retrieved from KEGG [50], [51]. Genes that are present in Helicosporidium are indicated by yellow boxes. Genes that are absent from Helicosporidium but present in Chlamydomonas are indicated by red boxes. Genes that are absent from both Helicosporidium and Chlamydomonas are indicated by empty boxes. (PNG) [file pgen.1004355.s010.png]

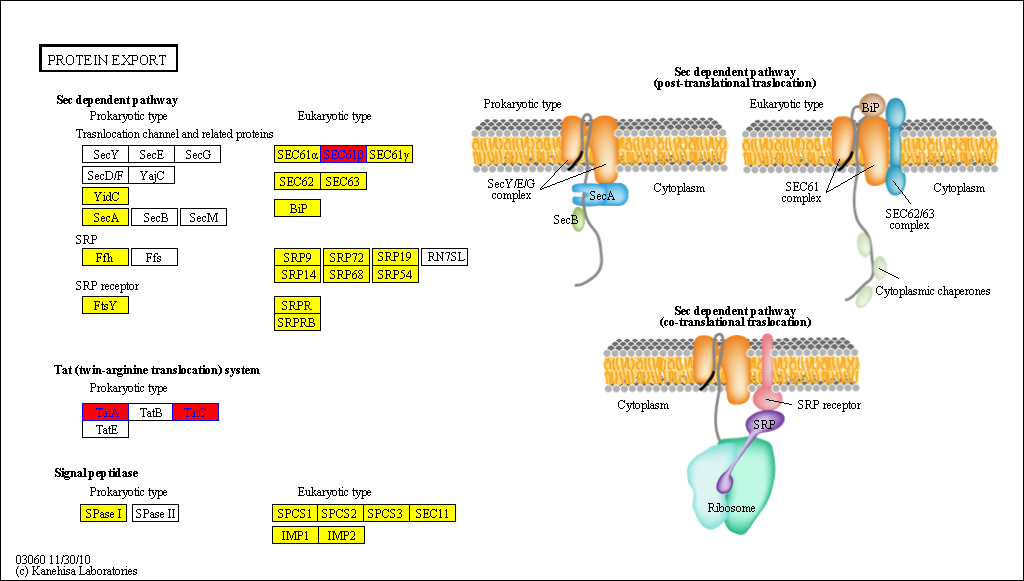

Supplement: Data S11 — Orthology map of the protein export pathways (ko03060) retrieved from KEGG [50], [51]. Genes that are present in Helicosporidium are indicated by yellow boxes. Genes that are absent from Helicosporidium but present in Chlamydomonas are indicated by red boxes. Genes that are absent from both Helicosporidium and Chlamydomonas are indicated by empty boxes. (PNG) [file pgen.1004355.s011.png]

U

C

T

P

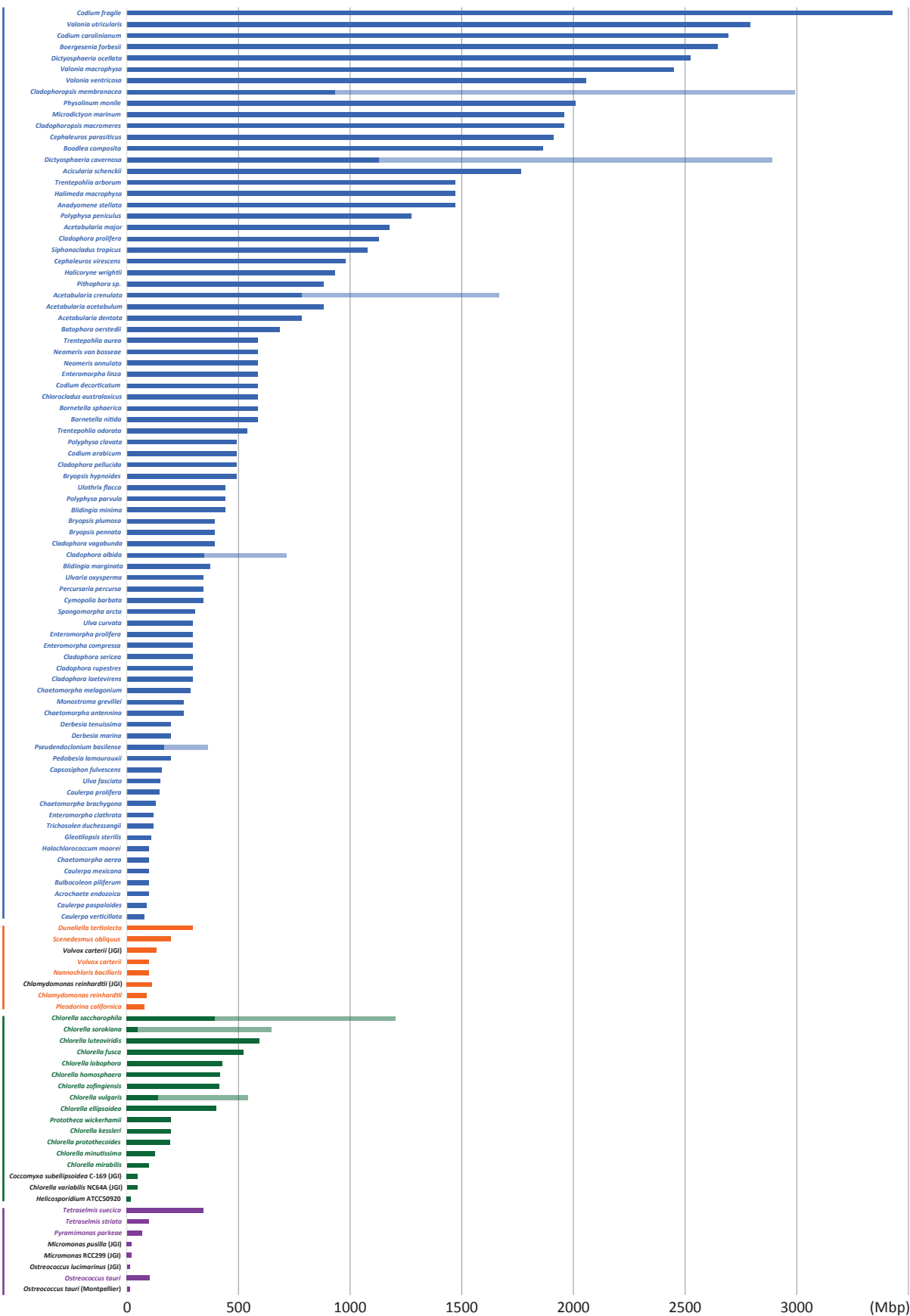

Supplement: Figure S1 — Green algal genome sizes in the phylum Chlorophyta. Sequenced green algae are labelled in black with the source indicated between parentheses. Estimated values based on nuclear DNA content from Kapraun [61], [62] are color-coded according to their respective group. Blue, U, Ulvophyceae; Orange, C, Chlorophyceae; Green, T, Trebouxiophyceae; Purple, P, Prasinophyceae. Lower and upper estimates, when present, are shown in dark and light colors, respectively. Note that the real and estimated sizes of the Ostreococcus tauri genome differ by an order of magnitude. (PDF) [file pgen.1004355.s015.pdf]

## Heme synthesis

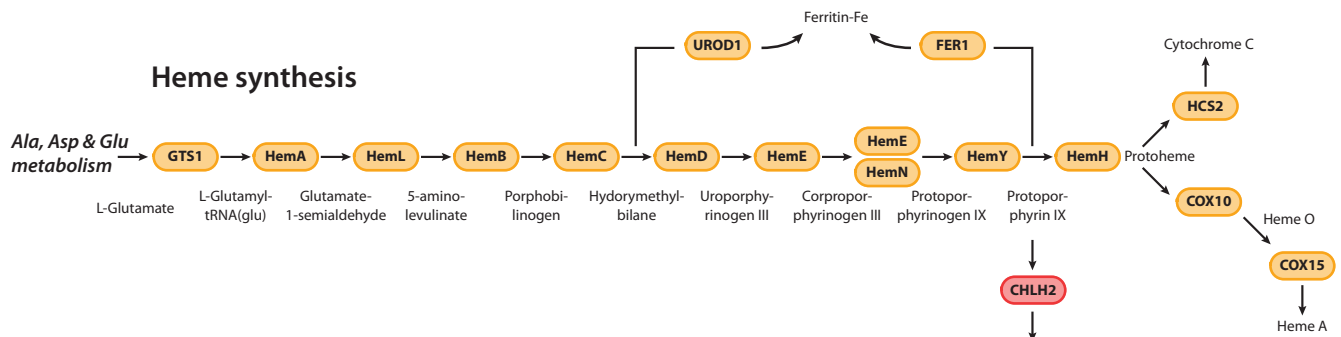

## Chlorophyll biosynthesis

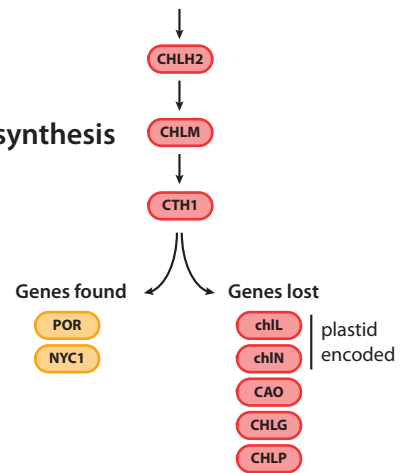

Supplement: Figure S2 — Heme and chlorophyll pathways in Helicosporidium. Genes present in Helicosporidium are indicated in orange. Genes absent are shown in red. This simplified schema is derived from KEGG pathway KO00860 [50], [51]. (PDF) [file pgen.1004355.s016.pdf]

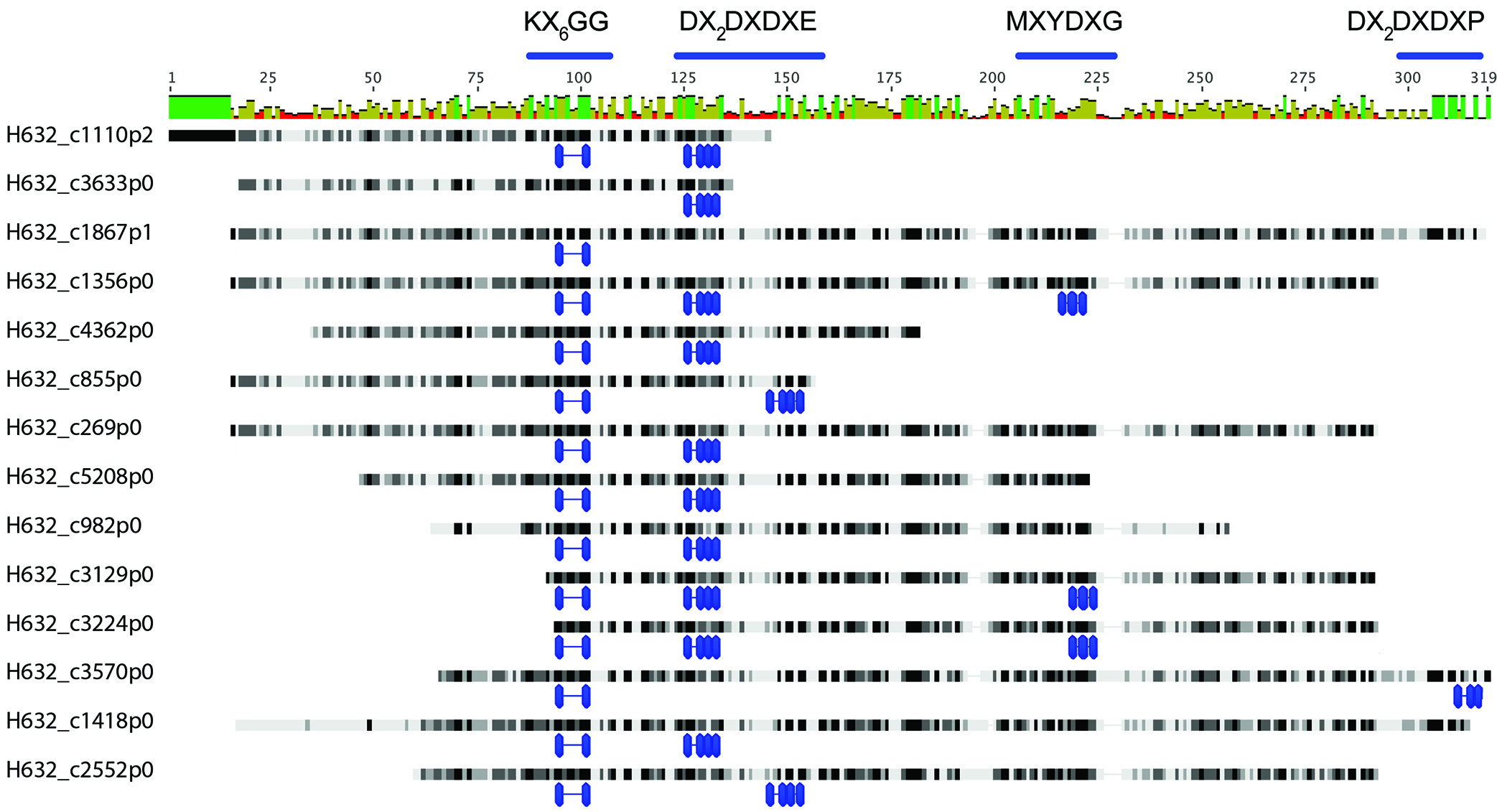

Supplement: Figure S3 — CLUSTALW alignment of the Helicosporidium chitinase Gly18 catalytic domains. Conserved motifs are shown in blue. BLOSUM, Gap open penalty: 35, Gap extend penalty 0.75. (TIFF) [file pgen.1004355.s017.tif]
